# Mouse Models of *c-myc* Deregulation Driven by IgH Locus Enhancers as Models of B-Cell Lymphomagenesis

**DOI:** 10.3389/fimmu.2020.01564

**Published:** 2020-07-23

**Authors:** Melissa Ferrad, Nour Ghazzaui, Hussein Issaoui, Jeanne Cook-Moreau, Yves Denizot

**Affiliations:** Inserm U1262, UMR CNRS 7276, Equipe Labellisée LIGUE 2018, Université de Limoges, Limoges, France

**Keywords:** MYC, B-cell lymphoma, transgenic mouse models, IgH locus, IgH transcriptional enhancers

## Abstract

Chromosomal translocations linking various oncogenes to transcriptional enhancers of the immunoglobulin heavy chain (IgH) locus are often implicated as the cause of B-cell malignancies. Two major IgH transcriptional enhancers have been reported so far. The E_μ_ enhancer located upstream of the C_μ_ gene controls early events in B-cell maturation such as VDJ recombination. The 3' regulatory region (3'RR) located downstream from the C_α_ gene controls late events in B-cell maturation such as IgH transcription, somatic hypermutation, and class switch recombination. Convincing demonstrations of the essential contributions of both E_μ_ and 3'RR in B-cell lymphomagenesis have been provided by transgenic and knock-in animal models which bring the oncogene *c-myc* under E_μ_/3'RR transcriptional control. This short review summarizes the different mouse models so far available and their interests/limitations for progress in our understanding of human *c-myc*-induced B-cell lymphomagenesis.

## Introduction

**Figure 1:**
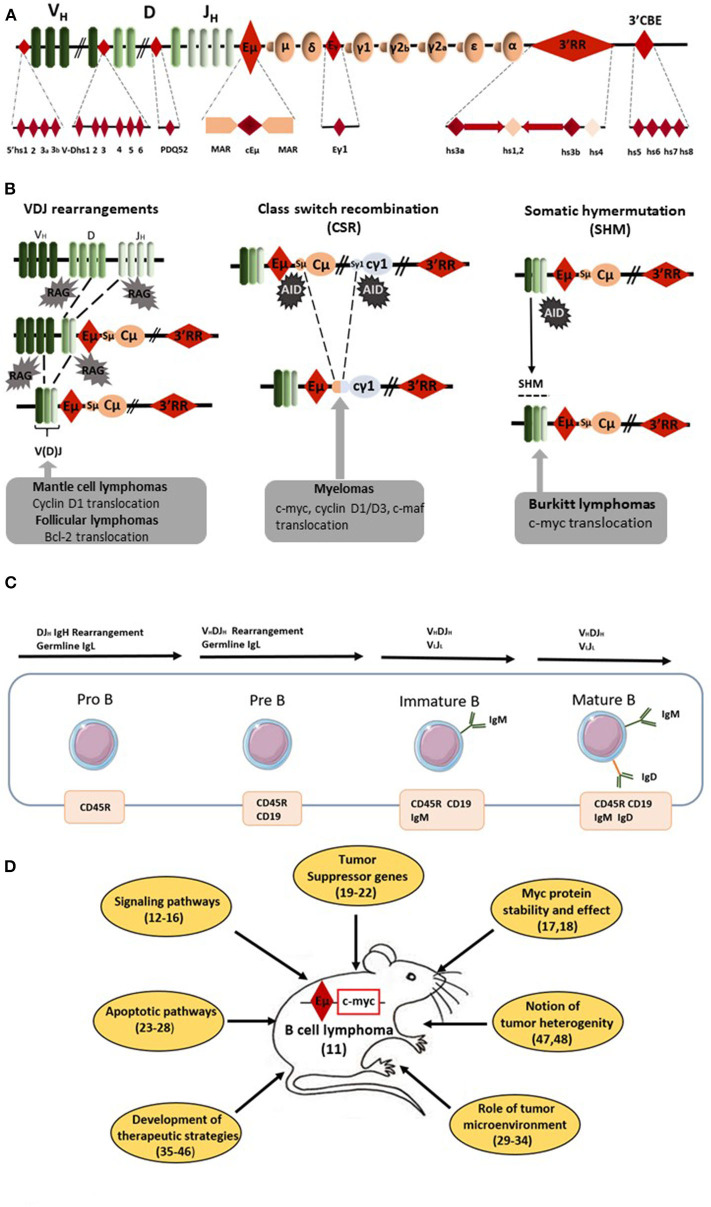
E_μ_-*Myc* mice as a model of B-cell lymphomagenesis. **(A)** Schematic diagrams of the mouse IgH locus. Locations of the various IgH *cis*-regulatory elements with enhancer or insulator activity are reported: four hs sites located 5' of the first V segments, six hs sites in the V–D intergenic region, the DQ52 promoter-enhancer, the E_μ_ enhancer (the core region (cEμ) and its flanking MARs), the γ1 enhancer, the 3' regulatory region (3'RR) [four enhancers (namely hs3a, hs1,2, hs3b, and 4) with flanking inverted repeats] and the 3'CBE insulator region (hs5, 6, 7, and 8) as the 3' boundary of the locus. **(B)** Schematic representation of oncogene translocation affecting the IgH locus during VDJ recombination, CSR and SHM. Arrows indicate the site of oncogene translocation found during follicular lymphomas, mantle cell lymphomas, myelomas, and Burkitt lymphomas. **(C)** Schematic representation of B-cell development from pro-B to mature B-cells. Lymphomas from E_μ_-*Myc* mice are from the pre-B to the mature B-cell stages. The immature B-cell stage is characterized by the expression of membrane IgM whereas membrane IgD occurs at the mature B-cell stage. **(D)** Schematic representation of the various field of research developed with E_μ_-*Myc* mice. Bibliographic references are reported (number in parenthesis).

RAG-induced recombination, AID-induced DNA breaks and mutations throughout B-cell development make the IgH locus a hotspot for translocations (1) (Figures 1A,B). Bcl-2 translocation, the typical hallmark of follicular lymphomas (FL), occurs during RAG-induced VDJ recombination. Cyclin D1 translocation, associated with mantle cell lymphomas (MCL), occurs either during AID-induced somatic hypermutation (SHM) or AID-induced class switch recombination (CSR). *C-myc* translocation, the typical hallmark of Burkitt lymphoma (BL), takes place during AID-induced SHM and CSR. Finally, several translocations (such as *c-myc, c-maf*, *cyclin D1/D3*) found in myelomas are also related to AID-induced CSR. During CSR, AID-induced DNA double strand breaks (DSB) appear in the switch (*S*) donor region (usually S_μ_) and in the *S* acceptor region (for example S_γ1_ and S_α_ for CSR toward IgG1 and IgA, respectively). *S* regions are of various lengths (for example 3.5 and 10 kb long for S_μ_ and S_γ1_, respectively) and are unusually G-rich. AID deaminates C into U at preferential AID hotspot motifs located throughout *S* regions. The AID-introduced U in *S* region DNA is removed by UNG to generate an abasic site that is recognized by the endonuclease APE1 generating a nick. A closely spaced, similarly created nick on the opposite strand induces a staggered DSB. Translocation of the DNA fragment encompassing *c-myc* is due to an off target AID effect on the chromosome bearing *c-myc*. Since AID transforms C to U all along *S* donor/acceptor regions, there is no common breakpoint identified in *S* regions for mature B-cell lymphomas. It is the same AID effect for SHM where AID targets the VDJ rearranged segments (and up to several kB in 3') and can induce DNA DSB for *c-myc* translocation. Similarly to CSR, there is no common breakpoint established in VDJ regions for mature B-cell lymphomas. During VDJ recombination RAG binds to recombination signal sequences adjacent to V, D, and J coding segments and induces DNA DSB. *C-myc* translocation could take place during this process. Similarly to CSR/SHM, there is no common breakpoint singled out in VDJ regions for B-cell lymphomas. The common point for all these c-myc translocations is the occurrence of DSB in the IgH locus during its remodeling required for B-cell repertoire formation and B-cell maturation. All remodeling events of the IgH locus (VDJ recombination, SHM, and CSR) require transcription to occur (2). Transcriptional control and remodeling of the IgH locus are under the control of several *cis*-regulatory elements located throughout the IgH locus. In the murine IgH locus seven regions of interest can be defined including *cis-*regulatory elements, matrix attachment regions (MARs), and hypersensitivity (hs) sites with potential transcriptional enhancer or insulator activity: 4 hs sites located 5' of the first V segments, 6 hs sites in the V–D intergenic region, the DQ52 promoter–enhancer, the E_μ_ enhancer (between J_H_ and *C*_μ_) and its flanking MARs, the γ1 enhancer element, the 3' regulatory region (3'RR) downstream from C_α_ with its four enhancers (hs3a, hs1,2, hs3b, and 4) and the 3'CBE insulator region (hs5, 6, 7, 8) as the 3' boundary of the locus (Figure 1A). Two potent transcriptional enhancers act during B-cell maturation: E_μ_ (during early B-cell maturation stages) and 3'RR (during late B-cell maturation stages) (Figure 1A). These elements obviously intervene in oncogene-induced B-cell lymphomagenesis as reported by several transgenic mouse models (using both transgene and knock-in (KI) strategies) developed in order to mimic human mature B-cell lymphomagenesis. Since c-myc is a key regulator of cell growth through its action on cell cycle progression, metabolism, differentiation, death receptor signaling, and DNA damage recovery, the vast majority of available models use *c-myc* as a deregulated oncogene (3). This short review describes how E_μ_ and 3'RR enhancers might play a critical role in *c-myc* deregulation during c-myc-induced mature B-cell lymphomas, why these models are not silver bullets to totally mimic human B-cell lymphomagenesis and why it is possible that targeting the 3'RR would be an interesting strategy in human B-cell lymphomagenesis.

## The E_μ_ *cis*-Transcriptional IgH Enhancer and *c-myc* Deregulation

Forty years ago, E_μ_ was the first discovered IgH *cis*-transcriptional enhancer (4–6). It is located upstream of the C_μ_ gene (Figure 1A). E_μ_-deficient mice revealed its role in controlling IgH locus access at immature B-cell stages and thus its key role for efficient VDJ recombination (7, 8). In contrast, E_μ_ is dispensable for late B-cell maturation events such as IgH locus transcription for Ig synthesis and CSR (9, 10). In 1985, transgenic mice bearing *c-myc* coupled to the E_μ_ enhancer were reported to consistently develop immature (pre-B) and sometimes mature B-cell lymphomas (11). Our entire knowledge of E_μ_ involvement in *c-myc* oncogenic deregulation for B-cell lymphoma development was built from this model. Since 1985, 183 papers with “E_μ_-*Myc* mice” in their abstract have been referenced. Of note, 153 have been published in the last 15 years showing the great interest of the scientific community for this transgenic mouse model of B-cell lymphoma. It is thus impossible in this short review to reference them all. Therefore, the authors apologize in advance for the numerous interesting manuscripts which have not been cited in the present review. Lymphomas from Eμ-Myc mice range from the pre-B to the mature B-cell stages (Figure 1C). They are usually all positive for the CD45R (B220), CD19 and CD93 (AA4.1) B-cell specific markers and negative for the CD3 T-cell marker. Tumors of pre-B-cell type are characterized by the lack of membrane IgM and no Ig light chain (IgL) rearrangements. Tumors of immature B-cell types are more mature and express membrane IgM after efficient IgL rearrangements. Tumors of mature B-cell types are even more mature and express both membrane IgM and IgD. The majority of lymphomas in Eμ-Myc mice are at the pre-B and immature B-cell stages. In their original study, Adams et al. (11) stated that “these *myc* mice should aid study of lymphoma development, B-cell ontogeny and Ig regulation.” Clearly 35 years later this is the case. Creation of these mice resulted in the dissection of many mechanisms implicated in B-cell lymphomagenesis (Figure 1D). They have highlighted the importance of several signaling pathways (such as Ras/Mapk, mTOR, and Akt) (12–14), several cell cycle check-points (such as Mdm2/p53/p73) (15, 16) and processes that affect c-myc stability and action (17, 18). Using these mice clearly demonstrated the importance of numerous (new and well-known) tumor suppressor genes (such as FoxO3, CDK4, Mtap, and Smchd1) (19–22). This model reinforced our knowledge concerning the signaling/regulation of the B-cell apoptotic program (members of the Bcl-2 family of apoptosis regulator) and deficiencies in apoptotic pathways leading to B-cell lymphomagenesis (23–28). To our knowledge the influence of genetic background in the development of B-cell lymphomas in Eμ-*Myc* mice has not been documented. The E_μ_-*Myc* model has also opened a new area of research concerning the role of tumor microenvironment via release of angiocrine/chemokine factors (29–31) and the importance of cells from the vascular niche for NK cell surveillance, senescence, and homing of B-cell lymphomas (32–34). Perhaps most importantly, this model is at the origin of a wide number of publications investigating new therapeutic treatments or combinations of drugs in order to affect (among various targets) DNA synthesis (cytarabine, doxyrubicin, cyclophosphamide), mTOR signaling (rapamycin analogs), microtubule formation (vincristine), c-myc (decursin), apoptosis (venetoclax and BET inhibitors), protein synthesis (silvestrol), or B-cell receptor (BCR)-induced, or chemokine-mediated signaling (ibrutinib) (35–42). The rapid occurrence of lymphoma in E_μ_-*Myc* mice and its high penetrance make this mouse model an accurate, reliable, easy, and fast experimental model not only to test new therapeutic approaches but also combinatory associations. This model is also unique by providing the possibility to monitor the assay of new NK therapeutic vaccination strategies (43, 44), to stimulate immune defenses for tumor rejection (45) and to test protocols for monoclonal antibody therapies (46). E_μ_-*Myc* mice have thus proven their great potential as a model to study human B-cell lymphomagenesis during the past decade. Moreover, arising lymphomas are heterogeneous (47, 48) mirroring genomic differences observed between human BL, germinal center B-cell lymphomas (GCBCL), activated B-cell lymphomas (ABCL), and diffuse large B-cell lymphomas (DLBCL). The different genomic signatures (toward specific proliferative and/or apoptotic pathways) of B-cell lymphomas in E_μ_-*Myc* mice might be used as biomarkers of response against specific therapeutic strategies. Thus, and especially with the development of transcriptomic tools, E_μ_-*Myc* mice can serve as relevant model for human B-cell lymphoma subtype experimental or associated treatments. The only but nevertheless major drawback of E_μ_-*Myc* mice relates to the window of activity for E_μ_ which has been clearly demonstrated to occur at the immature pro-B/pre-B B-cell stages (49, 50). E_μ_ is not implicated in IgH hypertranscription occurring at the mature/plasma cell stages. E_μ_ is also not implicated in DNA breaks occurring during SHM/CSR and thus clearly not implicated in oncogenic translocation induced by off target AID action occurring during CSR or SHM in the majority of human mature B-cell lymphoma subtypes. As confirmation of this fact, the great majority of lymphomas from E_μ_-Myc mice have a pre-B/immature B-cell stage.

## The 3'RR *cis*-Transcriptional IgH Enhancer and *c-myc* Deregulation

**Figure 2:**
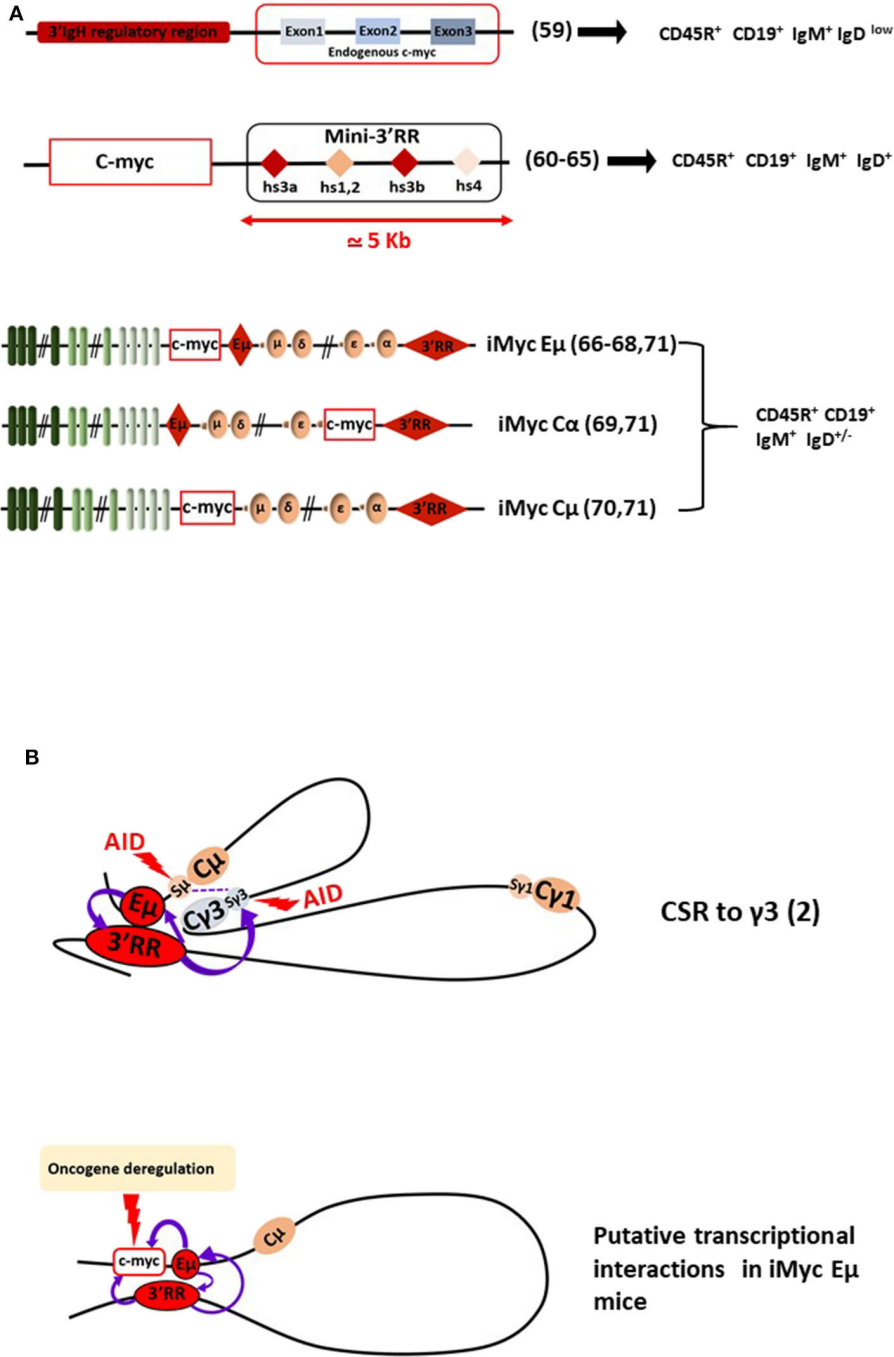
The 3'RR and B-cell lymphomagenesis. **(A)** Schematic representation of several transgenic mouse models reporting *c-myc* 3'RR-driven deregulation leading to B-cell lymphomagenesis. B-cell lymphoma phenotypes are reported. Bibliographic references are reported (number in parenthesis). The “Mini-3'RR” contains the four transcriptional enhancers hs3a, hs1,2, hs3b, and hs4 but not the 3'RR palindromic sequences flanking hs1,2 and the DNA sequence between hs3a and hs4. **(B)** Long-range loop interactions between chromatin segments of the IgH locus comprise the mechanism of normal gene transcription regulation by the E_μ_ and 3'RR transcriptional enhancers. The example of the IgG_3_ CSR process is schematized. Putative long-range interactions leading to c-myc oncogene deregulation in iMycE_μ_ mice are schematized.

The second transcriptional enhancer located in the IgH locus is the 3'RR (Figure 1). The 3'RR is a complex element with four transcriptional enhancers (namely hs3a, hs1,2, hs3b, and hs4) encompassed in a unique and functional 3D palindromic architecture (51). The 3'RR controls μ transcription (7), CSR (52, 53), and SHM (54) in mature B-cells. The transcriptional activity of the 3'RR occurs from pre-B to mature B-cell stages (55) and thus has a much larger window of activity than the E_μ_ enhancer. In 1994, Madisen and Groudine reported (in stable transfection assays in plasmacytomas and BL cells) that the 3'RR was efficient and sufficient to deregulate c-*myc* transcription (56). Convincing demonstration of 3'RR involvement in lymphomagenesis has been produced by a transgenic 3'RR-deficient model of B-cell lymphomas with IgH-*c-myc* translocations (57). The integrity of the 3'RR (deletion of hs3b to hs4) has been shown to be dispensable for development of pro-B-cell lymphomas with V(D)J recombination-initiated translocations suggesting the key role of E_μ_. In contrast, 3'RR integrity (for its optimal transcriptional activity) is required for B-cell lymphomas with CSR-associated translocations (57). In another study modeling murine plasmacytomas with T (12, 15) translocations, the same hs3b-hs4 deletion of the 3'RR in Bcl-xL transgenic mice was without effect for *Myc* deregulation and mouse plasmacytoma generation (58). However, total 3'RR deletion in these plasmacytomas lowered Myc expression and cell growth confirming 3'RR involvement for myc deregulation by T (12, 15). Nevertheless, these models are not sufficient to monitor in detail and to modulate signaling pathways for B-cell lymphoma development. The same comments can be made for the transgenic mouse model of Wang and Boxer (59) which develops mature B-cell lymphomas (CD19^+^B220^+^*IgM*^+^*IgD*^low^) after the KI of a 3'RR cassette upstream of the endogenous *c-myc* gene (this model is the reverse of natural *c-myc* translocation into the human IgH locus) (Figure 2). More than 15 years after the development of transgenic E_μ_-*Myc* mice, transgenic *Myc*-3'RR mice were generated and were shown to develop BL-like proliferations and diffuse anaplastic B-cell lymphomas (60). All these lymphomas exhibited a mature B-cell phenotype (CD19^+^B220^+^*IgM*^+^*IgD*^+^) but differed by their Ki67 status (low and high for diffuse anaplastic B-cell lymphomas and BL lymphomas, respectively). This model was used to study the role of second hits such as p53 deficiency, Cdk4 mutation, and change of class-specific B cell receptor (BCR) tonic signals. Results clearly demonstrated that a second hit affects the phenotype of B-cell lymphomas, their aggressiveness and transcriptomic signatures differently (61–64). This model was, however, progressively abandoned due to its medium B-cell lymphoma penetrance (compared to E_μ_-*Myc* mice), long delay for B-cell lymphoma development (compared to E_μ_-*Myc* mice), key differences with human B-cell lymphomas (such as mutations lacking for the p53-ARF-Mdm2 apoptotic pathways in numerous cases) and the description that the occurrence of B-cell lymphomas was much too sensitive to genetic background [C57Bl/6 mice developed BL-like lymphomas while none occurred in a Balb/c background (65)]. All these points argued against the use of *Myc*-3'RR mice as an accurate experimental model to test new pharmacologic or vaccination strategies.

## The Combination of E_μ_ and 3'RR *cis*-Transcriptional Enhancers and *c-myc* Deregulation

As reported above, a transgenic model with IgH-*c-myc* translocations in response to pristine demonstrated the involvement of IgH *cis*-transcriptional enhancers in B-cell lymphomagenesis (57). In another manner, this study confirmed results obtained with three transgenic mouse models with a *c-myc* KI in various locations in the IgH locus (i.e., under the dependence of both E_μ_ and 3'RR elements) (Figure 2). These models provided the most convincing data for the essential roles of both E_μ_ and 3'RR in c-*myc* B-cell lymphomagenesis. The KI of *c-myc* in the mouse IgH locus just 5′ to E_μ_ (namely i*Myc*E_μ_ mice), thus modeling human endemic BL, induced, as expected, B-cell lymphoma development with alterations in the p19^Arf^-Mdm2-p53 tumor suppressor axis (66) and NF?B/STAT3/PI3K signaling (67). In this model, *c-myc* is under the control of both E_μ_ and 3'RR at immature and mature B-cell stages, respectively. i*Myc*Eμ mice also mimic T (12, 15) mouse plasmacytoma translocation and thus also lead to plasmacytomas (68). KI of *c-myc* directly into C_α_ just 5' to the 3'RR (i*Myc*C_α_ mice) produced B-cell lymphomas with low kinetics which were increased after overexpression of the anti-apoptotic Bcl-X_L_ gene (69). In this model, *c-myc* is located in a site where E_μ_ has no transcriptional influence, *c-myc* transcription being only under the dependence of 3'RR at mature B-cell stages. *c-myc* KI in the mouse IgH locus just 5′ to C_μ_ with E_μ_ deletion (namely i*Myc*C_μ_ mice), thus modeling human sporadic BL, confirmed that 3'RR alone is sufficient to deregulate *c-myc* in the B-cell lineage and to induce B-cell lymphoma development (70). Taken altogether, these KI models carrying *c-myc* at the IgH locus are prone to B-cell lymphomas of various penetrance, kinetics, and fate as recently reported in a study comparing the three mouse models (71). The lymphoma signatures are also heterogeneous even comparing lymphomas from a specific KI, mirroring the genomic differences observed between the various subtypes of human mature B-cell lymphomas and those previously reported with the model of transgenic E_μ_-*Myc* mice. In our opinion, these transgenic mouse models represent the “most physiological” experimental mouse models by mimicking the direct effect of c-*myc* in the context of the endogenous IgH locus. However, the main drawbacks of these various KI mice (and similarly to *Myc*-3'RR mice) remain their low lymphoma penetrance and their low kinetics of B-cell lymphoma development arguing against their use as efficient and easy experimental models to test new experimental therapeutic approaches. The low kinetics of B-cell lymphoma development compared with 3'RR-*Myc* mice would be related to the 3'CBE insulator region at the 3' boundary of the endogenous IgH locus (72, 73). This region is not present in the transgenic mouse model of 3'RR-induced *c-myc* deregulation. The 3'CBE insulator region contains a high density of binding sites for CCCTC-binding factor (CTCF), a protein associated with mammalian insulator activity. Deletion of the 3'CBE insulator region resulted in significant effects on VDJ rearrangement, IgH locus compaction, and IgH locus insulation. Furthermore, physical interactions occur in B-cells between 3'CBE and 3'RR enhancers suggesting that the entire 3' region (3'RR enhancers + 3'CBE insulators) works as a physical unit. The lack of 3'CBE in 3'RR-*Myc* mice could induced stronger and longer ***c****-myc* deregulation (and thus faster lymphoma emergence) than that obtained when *c-myc* is inserted into the IgH locus under the control of the entire (enhancer + insulator) region.

## Conclusion

Knock-out mice models have clarified the functions of E_μ_ and 3'RR enhancers as essential for DNA remodeling and IgH locus transcription at specific stages of B-cell development and maturation. Thus, these enhancers have a major potential to be oncogene deregulators for IgH-translocated oncogenes, even when the breakpoints lie several 100 kb away from them. All these models contribute different but interesting data to our understanding of human B-cell lymphoma development and treatments especially with regards to the great functional and structural similarities found between human and mouse IgH loci (74). However, we must keep in mind that these mice are experimental models that do not reflect 100% of what happens in humans. For example, if the vast majority of human mature B-cell lymphomas are mutated in their VDJ region (highlighting their post-germinal center status) it is not the case in mice where mature B-cell lymphomas are unmutated (highlighting their pre-germinal center status) (75). Long-range loop interactions between chromatin segments of the IgH locus comprise the mechanism of normal and abnormal gene transcription regulation by the 3'RR (2, 76) (Figure 2B). Therefore, it is possible to suggest that targeted inhibition of the 3'RR would be a therapeutic approach for the treatment of some mature B-cell lymphomas. Finally, it is also of importance to mention that the *c-myc* oncogene driven by Ig light chain enhancers also induces B-cell lymphoid malignancy in transgenic mice (11, 77). These models highlight not only the importance of all Ig enhancers for B-cell lymphoma development but also that a 3'RR targeting strategy (if any) would not be a silver bullet to treat all B-cell lymphomas but at best some mature B-cell subtypes.

## Author Contributions

All authors listed have made a substantial, direct and intellectual contribution to the work, and approved it for publication.

## Conflict of Interest

The authors declare that the research was conducted in the absence of any commercial or financial relationships that could be construed as a potential conflict of interest.
